# Surgical Repair of Spontaneous Lung Herniation Induced by Vigorous Coughing: A Case Report and Literature Review

**DOI:** 10.7759/cureus.37325

**Published:** 2023-04-09

**Authors:** Vasileios Leivaditis, Konstantinos Grapatsas, Athanasios Papatriantafyllou, Efstratios N Koletsis, Nikolaos Charokopos, Manfred Dahm

**Affiliations:** 1 Department of Cardiothoracic and Vascular Surgery, Westpfalz-Klinikum, Kaiserslautern, DEU; 2 Department of Thoracic Surgery and Thoracic Endoscopy, University Medicine Essen – Ruhrlandklinik, Essen, DEU; 3 Surgery, General Hospital of Patras, Patras, GRC; 4 Department of Cardiothoracic Surgery, Patras University Hospital, Patras, GRC

**Keywords:** intercostal space rupture, thoracic wall weakness, chest wall herniation, spontaneous lung hernia, lung herniation

## Abstract

Lung herniation is a rare clinical entity defined by extrathoracic protrusion of the lung or lung tissue due to a weakness in the thoracic wall. We present here a case of a 72-year-old male who presented with a spontaneous lung herniation, which occurred as a result of a ventral luxation of the third rib from the sternocostal joint due to vigorous coughing. The defect was repaired through anterolateral thoracotomy, reposition of the lung and approximating the ribs using heavy sutures. The postoperative course of the patient was uncomplicated. A brief review of the literature is also provided.

## Introduction

Lung hernias are relatively uncommon conditions that occur when a portion of the lung or lung tissue protrudes through a weak or damaged area of the chest wall. These hernias can be caused by a variety of factors, including trauma, surgery, and underlying medical conditions. Roland was the first to describe this clinical condition in 1499 [1]. Spontaneous lung hernias are even more rare conditions where the same phenomenon occurs without any known traumatic cause. These hernias are thought to result from underlying medical conditions that weaken the thoracic wall, such as chronic obstructive pulmonary disease (COPD) or emphysema [2]. Despite their relative rarity, spontaneous lung hernias can have significant impacts on a patient's respiratory function and overall health. Cases of spontaneous lung hernias have been little reported in the existing literature [2,3].

## Case presentation

A 72-year-old male patient with a history of smoking, COPD and arterial hypertension was admitted to the emergency department of our hospital due to progressive severe pain of the left hemithorax and shortness of breath. Physical examination revealed a bulge in the left anterior chest wall and decreased breath sounds in that area, accompanied with a pronounced hematoma of the left flank. No history of trauma was reported. He had already been treated about four weeks ago due to persistent non-productive cough in the emergency room. At that time a small subcutaneous ecchymosis had been detected in this area.

**Figure 1:**
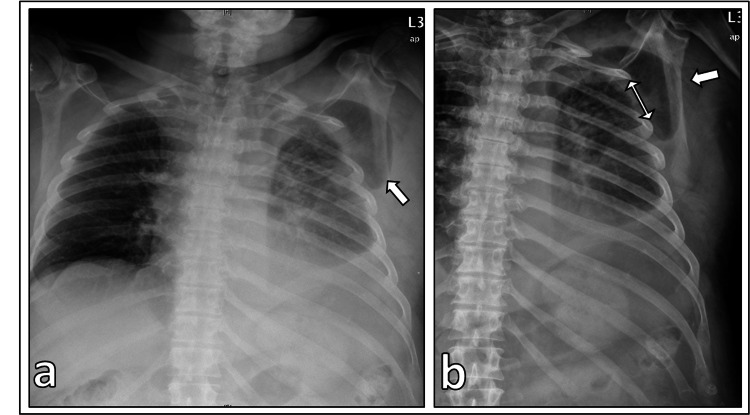
X-ray images X-ray image is showing the extrathoracic lung protrusion (arrow) and the thoracic wall defect between the ribs (double arrow). a. thorax, b. left hemithorax.

**Figure 2:**
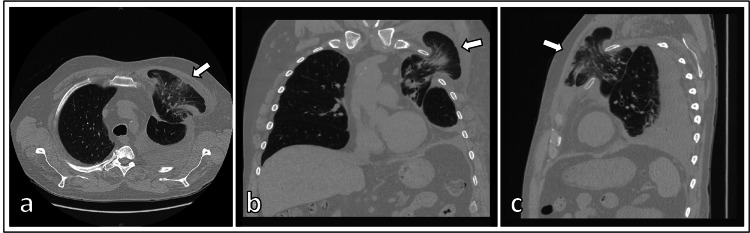
Computed tomography images The images are showing the lung hernia (arrow). a. transversal view, b. coronary view and c. sagittal view.

The chest X-ray showed a left-sided pleural effusion with a pulmonary prolapse through the third left intercostal space (Figure 1). No signs of pneumothorax were detected. Computed tomography (CT) was also performed and showed a pulmonary protrusion on the left hemithorax with prolapse of parts of the left upper lobe through the third intercostal space outside the thoracic wall with ventral luxation of the third rib in the costosternal joint (Figure 2). There were also bleedings with dystelectases in the prolapsed lung parenchyma. An accompanying thoracic tissue hematoma was also present on the left side of the thorax. A hemorrhagic pleural effusion with a maximum width of approximately 4 cm was also detected. No signs of pneumothorax or sternum and rib fracture were detected.

Further evaluation with pulmonary function tests revealed reduced lung capacity and restricted airflow. Based on the patient's symptoms and imaging results, a diagnosis of spontaneous lung hernia was made. The patient was referred to our department for surgical management. Echocardiography was also performed and revealed a normal left and right ventricular function, a normal ejection fraction and no wall motion disorders or any valve disease. The patient was overall in a good general condition, and there was no contraindication for surgical treatment. The patient was not on any anticoagulation treatment.

**Figure 3:**
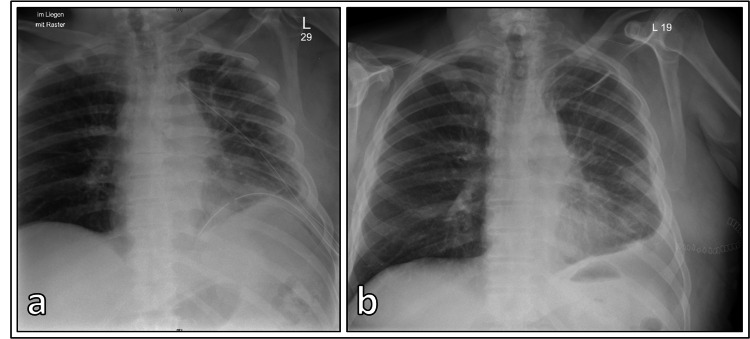
X-ray images after the surgery a. Direct postoperative X-ray after repair and b. after the removal of the chest drains.

The patient underwent open surgical repair of the hernia through a small anterolateral thoracotomy. The left upper lobe was successfully totally repositioned in the pleural cavity without the need for any lung parenchyma resection. The intercostal space was then closed with Vicryl sutures, including a costochondral joint reapproximation. The surgery was successful, and the patient's chest pain and shortness of breath resolved following the procedure (Figure 3). The patient was postoperatively successfully early mobilized and received intensive physiotherapy.

The patient was started on a rehabilitation program to improve lung function and prevent further complications. He was also treated for his underlying COPD, including pharmacotherapy and respiratory therapy. Follow-up re-evaluation with CT scans and spirometry after three months showed improvement in the patient's lung function and no evidence of recurrent herniation. The patient remained asymptomatic, free of pain, and with no limitations in his daily activities.

## Discussion

This case report highlights the importance of early recognition and prompt treatment of spontaneous lung hernias. The patient's presentation of chest pain and shortness of breath, combined with the physical examination findings and imaging results, helped to make the diagnosis of spontaneous lung hernia. The successful surgical repair of the hernia and rehabilitation program to improve lung function helped to prevent further complications and improve the patient's overall health and life quality. Spontaneous lung hernias are as mentioned before very uncommon conditions. Their exact incidence remains unclear and has probably not been fully investigated yet [2].

Causes of lung hernias

Lung hernias can be caused by a number of different factors, including trauma, surgery, and underlying medical conditions. Traumatic lung hernias typically occur as a result of blunt force trauma to the chest, such as from a car accident or a fall [4-7]. Surgical lung hernias can occur as a result of previous thoracic surgeries, such as a lobectomy or a pneumonectomy [4]. Certain underlying medical conditions, such as emphysema or chronic obstructive pulmonary disease (COPD), can weaken the lung and make it more susceptible to herniation [2,4]. The majority (80%) of acquired hernias occur as a consequence of trauma [7].

The exact cause of spontaneous lung hernias is not well understood, but it is believed to result from the combination of underlying medical conditions that weaken the chest wall and increase the risk of herniation [2]. Such conditions include COPD and emphysema, which cause damage to the lung tissue and result in decreased lung volume and increased pressure within the chest. This increased pressure can put stress on the chest wall, leading to herniation [1,2,8]. COPD patients may also be predisposed due to chronic coughing and hyperinflation, which also cause changes in the intrathoracic and intrabdominal pressures. Obesity is an additional risk factor that may accelerate the onset of the herniation [2,9,10]. Male gender and tobacco use are also considered as relevant risk factors [11]. Steroid use is also considered to be a significant risk factor, as it contributes to the weakening of the thoracic wall [2,7,8,11].

The underlying mechanism, although not having been clearly understood, has been described as depending on increased intrathoracic pressures and forces applied to the thoracic wall caused by severe cough or straining [10]. The most well know weak positions of the thoracic cage have been reported anteriorly near the sternum, medial to the costochondral junction, and posteriorly, near the vertebral bodies, where there is a single layer of intercostal muscles [5,10]. Strong bending forces applied on the middle third of the rib are considered to be the result of shearing forces in opposite directions of the serratus anterior and external oblique muscles [6,12]. In cases where spontaneous non-traumatic rib fractures are involved, the typical localisation of the fracture is mostly reported at the lateral or anterior parts of the rib, a fact which further supports the above theory [2].

Classification of lung hernias

The Morel-Lavallée classification is a system used to classify lung hernias, which divides lung hernias into subtypes based on their location and aetiology [2,13]. It was developed in 1845 and was based on 32 cases. It created a very comprehensive and, till now, the most widely accepted classification for lung hernias based on both their aetiology and anatomic location [14]. The types of herniation according to this classification are presented in Table 1.

**Table 1:** The types of spontaneous lung herniation according to the Morel-Lavallée classification.

Type of Classification	Type of Herniation
Anatomic Classification	1. Cervical / Supraclavicular
	2. Thoracic / Intercostal
	3. Diaphragmatic
	4. Mediastinal
Aetiological Classification	1. Congenital
	2. Acquired: (Traumatic, Spontaneous, Pathological, Postsurgical)

About 35% of all lung hernias are of cervical location. They occur when a part of the lung protrudes through the anterior region of the thoracic inlet and it usually develops between the anterior scalene muscle and the sternocleidomastoid muscle as a result of the weakness of the suprapleural membrane (Sibson's fascia) [3,15]. Thoracic of intercostal lung hernias are the most common and account for approximately 60%-80% of all lung hernias. They are, as mentioned before, a protrusion of the lung parenchyma through a zone of weakness in the chest wall between the ribs [15]. Traumatic hernias are the most often and can occur either immediately or years after the injury. Diaphragmatic and mediastinal lung hernias are rare [3,15]. Etiologically, about 18% of pulmonary hernias are congenital, 52% are post-traumatic, 29% are spontaneous, and 1% are pathological [3,15]. As far as congenital hernias are concerned, these are rare and mostly associated with costal or cartilage defects such as rib hypoplasia, intercostal hypoplasia, or weak endothoracic fascia [3,4,14]. They are diagnosed in childhood, but sometimes they may remain asymptomatic and present their first manifestation later in adult life [4,14]. Pathological hernias are associated with infectious or tumor pathologies which involve a part of the chest wall [3]. The Morel-Lavallée classification has been helpful in determining the severity of a lung hernia and plan appropriate treatment [2,13]. According to the anatomic classification of this system, the hernia in our reported case is one of the thoracic or intercostal type. According to the aetiological classification, our case belongs to the spontaneous subtype.

Another classification regarding the extension of the herniation and the need for repair can also be proposed. According to this classification, the lung hernia in our case is a type II of herniation. This is presented in Table 2.

**Table 2:** Classification of the lung herniation according to its extension and the requirement of surgical treatment.

Type of herniation	Description
Type I	Small hernias that involve only the parietal pleura (the lining of the chest wall) and do not affect the lung tissue itself. These hernias are usually asymptomatic and may be discovered incidentally on imaging studies.
Type II	Hernias that involve both the parietal pleura and the lung tissue. These hernias may cause respiratory symptoms, such as shortness of breath or coughing, and may require surgical treatment.
Type III	Large hernias that involve the entire lung and extend into the pleural cavity. These hernias can be life-threatening and require urgent surgical intervention.
Type IV	Hernias that are associated with other injuries or complications, such as chest trauma or infection.

Epidemiology of spontaneous lung hernias

The exact incidence of spontaneous lung hernias is unknown, as they are often misdiagnosed or undiagnosed due to their rarity and non-specific symptoms. However, they are estimated to occur in less than 1% of all patients with COPD or emphysema. Spontaneous lung hernias are more common in older individuals, and in those with a history of smoking or exposure to environmental toxins. Spontaneous hernias may also be associated with conditions that cause non-traumatic rib fractures [6,13]. The pathogenetic mechanism includes conditions of increased intrathoracic pressures during severe acute or chronic cough, sneezing, weightlifting, or straining [2,6,13]. About 120 cases have been reported in the current literature so far [2]. An apparently increasing number of reports has been, however, recorded in recent years [2].

Symptoms of lung hernias

The symptoms of lung hernias can vary depending on the severity of the hernia and the underlying cause. Common symptoms include chest pain, shortness of breath, and coughing. In some cases, a person may experience a persistent cough that produces blood or other fluids. Chest pain may be felt at the site of the hernia or elsewhere in the chest and can be sharp or dull [1,2]. Shortness of breath is typically more severe when the hernia is large and may be accompanied by rapid breathing. The main presenting findings of the physical examination may be bulging, with ecchymosis in the affected area and rarely signs of infection or incarceration [2]. Patients who suffer from lung herniation are potentially at risk of severe complications, some of which may become chronic [2]. The most important complications are presented in Table 3.

**Table 3:** The most important mid-term and long-term complications of lung herniation.

Complications
Incarceration and ischemia of the involved lung parenchyma
Acute respiratory distress syndrome (ARDS)
Systemic inflammatory response syndrome (SIRS)
Hemoptysis
Atelectasis
Pneumonia
Pleural effusion
Defect enlargement
Interstitial fibrotic parenchymal lesions
Chronic pain syndrome
Impaired quality of life

Diagnosis of lung hernias

Lung hernias are typically diagnosed through a combination of physical examination, medical imaging, and pulmonary function tests. A careful differential diagnosis must rule out other severe entities such as pulmonary embolism, pneumonia, malignancies, and abuse [2]. Other entities which are involved in the differential diagnosis are lipomas, delayed seromas, subcutaneous emphysema, bronchopleural fistulas, pectoralis major tendon rupture, abscess and metastases [14]. Chest X-rays and computed tomography (CT) scans are commonly used to visualize the chest wall and identify areas of herniation. Pulmonary function tests, such as spirometry, can help measure a person's breathing capacity and identify any restrictions in airflow. The diagnosis of spontaneous lung hernias can, however, be very challenging, as the symptoms are often non-specific and may be similar to other conditions [2,9]. Detorakis et al., in their case report, demonstrated the role of imaging, especially chest CT with multiplanar image reconstructions and maximum (MIP) and minimum intensity projection (MinIP) reformats [14]. Such imaging techniques are able to confirm the presence of the herniated lung, the hernial sac, the hernial orifice in the chest wall, and exclude possible complications such as lung tissue strangulation [14]. Thoracic ultrasound may also be useful in the diagnosis of pulmonary hernias, especially in cases when CT is not immediately available [3]. It has the advantage of being immediately available, accessible, non-irradiating and less expensive. It of course requires a certain experience of the person who performs it [3].

Treatment of lung hernias

The treatment of lung hernias depends on the underlying medical conditions, the size and location of the hernia, and the severity of the symptoms. A gold standard treatment for lung hernias has not been established yet [2]. There are no randomized controlled trials available in order to determine the superiority of conservative versus surgical treatment [1]. Data on the management of spontaneous lung hernias mainly derives from case reports and small case series. For small, asymptomatic hernias, observation and monitoring may be sufficient [6,9]. In cases of persistent pain or increasing hernia size, surgical intervention is recommended [1]. Moreover, in cases of hernias concerning the anterior thoracic wall, surgery is preferred over conservative treatment, irrespective of the size of the hernia, due to the potential extension into a thoracoabdominal hernia [1]. Surgical repair has been reported to be mostly achieved by thoracotomy at the level of the defect, with or without mesh application. Primary surgical closure options vary and can include wires, soft synthetic patches, rib plating, judet staples, and sliding staples [3]. The defect in the chest wall is directly sutured or closed using a mesh to reinforce the closure. This technique is typically used for small herniations or when the herniation is detected early [3]. Muscle flaps may also be used instead of synthetic mesh grafts. With the muscle flap closure technique, the muscle from the chest wall is used to cover the herniation and reinforce the closure. This technique is typically used for larger herniations or when there is a risk of recurrence [2,3]. Surgical closure options vary and can include wires, soft synthetic patches, judet staples, and sliding staples [3]. Muscle flaps may also be used instead of synthetic mesh grafts. No postoperative adverse events and very rare recurrences have been reported [2]. In severe cases, surgery is almost always required to repair the hernia and prevent further complications. Surgical options may include thoracoscopic repair, which involves making small incisions in the chest and using minimally invasive techniques to repair the hernia, or open thoracotomy, which involves making a larger incision in the chest to access the hernia and repair it. Thoracoscopic techniques are less invasive than open surgery and may result in less pain and a shorter recovery time [2,3]. In addition to surgery, treatment may also involve managing underlying medical conditions, such as COPD, to reduce the risk of herniation and improve overall respiratory function [2,9]. Smoking cessation, cough control, dietary hygiene, and functional rehabilitation are measurements that can improve the respiratory function and minimize the risk of recurrence [3]. Bronchoscopy may also be required in some cases in order to rule out a possible endobronchial lesion when such one may be suspected.

As mentioned before, increasingly more cases of spontaneous lung hernias have been reported in the literature during the last decade [1,2,7,11,16-19]. Ugolini et al. reported five patients, all of which were males with a median age of 68 years [2]. Herniations typically presented with lateral chest pain and a history of cough. A visible or palpable bulging was apparent in most patients (80%), as well as bruising of the surrounding area (60%). Respiratory symptoms were moderate, with shortness of breath requiring nasal flow oxygen occurring in 40% of the patients [2]. Normal X-ray was able to detect the lung hernia in only a 20% of the patients, whereas CT scan was diagnostic in 100% of the cases. They also present an algorithm for the management of such patients [2]. According to the proposed algorithm, in cases of patients with body mass index (BMI) >30, a 3-4-month trial of weight loss should be considered before scheduling the surgery if the patient does not require an urgent procedure [2]. In cases of patients under steroid therapy, elective surgery was advised if an urgent procedure was not necessary. Conservative treatment in such patients should not be considered due to the improbability of regression of the thoracic wall defect [2]. Sinopoli et al. presented a case series of 12 patients with spontaneous chest wall herniation and central obesity [9]. The failure rate of operative intervention in these patients was 66%. Notably, every patient who had a recurrence after their first surgical attempt at repair had a BMI ≥35. Obesity is therefore considered to be a significant contributor to chest wall herniation, especially when rib fractures occur. Patient selection is thus critical as far as the success of the surgical intervention is considered [9]. Hamid et al. reported a case of a middle-aged male with COPD exacerbation with severe bouts of cough, who developed a spontaneous lung herniation, which was initially managed conservatively, but later it progressed to pneumothorax and pneumomediastinum, with extensive subcutaneous emphysema [1]. Blowhole incisions were done on the anterior chest wall, which led to ultimate recovery. Kollipara et al. performed a literature search from January 1968 to April 2020 [17]. They identified 52 cases of spontaneous lung herniation. The patient population was predominantly male, with a median age of 58 years; 63.4% of the patients were current or previous smokers, and COPD was recorded in 48% of cases; 36% were described as obese with BMI; >30. 71% of the cases were associated with cough. Herniation in the left hemithorax was observed in 53% of the patients, localised posterior in 45% of the cases, and between the 8th and 9th intercostal space in 34.7% of the patients. The size of the herniation was >10 cm in most cases (75%). Previous thoracic surgery and rib fracture were documented in only a small number of patients. Forty-six patients received treatment, and 78% of them underwent primary surgical repair [17]. They also concluded that the presence of COPD or smoking is strongly associated with spontaneous lung herniation [17].

## Conclusions

Lung hernias are relatively uncommon conditions that can have significant impacts on a person's respiratory function and overall health. The causes of lung hernias are varied and can include trauma, surgery, and underlying medical conditions. Spontaneous lung hernias are even rarer clinical entities that can result from underlying medical conditions that weaken the chest wall, such as COPD or emphysema. Symptoms can include chest pain, shortness of breath, and coughing, and diagnosis is typically made through a combination of physical examination, medical imaging, and pulmonary function tests. Treatment options vary depending on the underlying cause and severity of the hernia and may include observation, monitoring, or surgical repair. In our case, a spontaneous intercostal hernia was successfully surgically treated and the patient postoperatively received suitable physical therapy and treatment of his underlying COPD with excellent long term results. Overall, early detection and prompt treatment of lung hernias is crucial for preventing further complications and improving respiratory function.
